# Differential effects of emotionally versus neutrally cued autobiographical memories on performance of a subsequent cognitive task: effects of task difficulty

**DOI:** 10.3389/fpsyg.2012.00299

**Published:** 2012-08-08

**Authors:** Kymberly D. Young, Kristine Erickson, Wayne C. Drevets

**Affiliations:** ^1^Section on Neuroimaging in Mood and Anxiety Disorders, National Institute of Mental Health, National Institutes of HealthBethesda, MD, USA; ^2^Laureate Institute for Brain Research, TulsaOK, USA; ^3^Department of Psychiatry, Oklahoma University College of Medicine, TulsaOK, USA

**Keywords:** autobiographical memory, cognition, task difficulty, episodic memory, emotion, rumination

## Abstract

Attention is a limited resource, and in order to improve processing of the attended information, competing processes must be suppressed. Although it is well established that an experimentally induced change in mood state comprises one type of competing process that can impair performance on a subsequent task, no study has investigated whether an emotionally valenced autobiographical memory (AM) also can alter performance on a subsequent task. We therefore examined the effects of AM recall on cognitive performance. Healthy participants (*n* = 20 per experiment) recalled AMs in response to positive, negative, and neutral cue words. Following each AM participants completed a simple perceptual task (Experiment 1) or solved moderately difficult subtraction problems (Experiment 2). In Experiment 1 participants performed less accurately following exposure to positive or negative versus neutral cue words (*ps* < 0.001), and also were less accurate following negative versus positive cue words (*p* < 0.001). In Experiment 2, in contrast, no difference in accuracy or response times reached statistical significance. Performance accuracy even trended toward being *higher* following exposure to negative versus neutral cue words (*p* = 0.08). The results of Experiment 1 suggested that recalling emotionally salient AMs reduces the attention directed toward a simple continuous performance task administered immediately following the AM task, conceivably due to persistent contemplation of the AM. The negative results of Experiment 2 suggested that the effect of AMs on attention was attenuated, however, by increasing the difficulty of the subsequent task. Our results have implications for patients with major depressive disorder (MDD), as performing cognitively demanding tasks may allow them to attenuate the impairing effects of negative rumination on cognition.

## Introduction

Attention is a limited resource, and in order to improve processing of the attended information, competing processes must be suppressed (Posner and Peterson, 1990; Posner, 1995). When such a competing process is not successfully suppressed, performance is impaired on subsequent attention-demanding tasks. An experimentally induced change in mood state is one such competing process that can affect attention and impair performance on a subsequent cognitive task (Martin and Kerns, 2011; Melcher et al., 2011). Research using mood induction techniques such as those that require participants to read statements intended to be elating or depressing (Velton, 1968) has shown that both positive and negative mood induction can impair performance on a variety of cognitive tasks (reviewed in Mitchell and Phillips, 2007). Impaired cognitive performance following positive mood induction has been demonstrated in tasks involving working memory as assessed using digit span, spatial planning as measured using the Tower of Hanoi task, and attention as evaluated using the Stroop task. Impaired performance following negative mood induction also has been reported on tasks of working memory and spatial planning.

Although it is well established in the literature that the induction of a change in emotional experience can impair performance on a subsequent cognitive task, no study has investigated whether eliciting an emotionally valenced autobiographical memory (AM) can alter performance on a subsequent task. The cognitive response that follows an emotionally evocative AM, whereby an individual contemplates the experience and its associated feelings, meanings, and consequences, forms one type of competing process that can interfere with the disengagement of attention to such an extent that the reallocation of attention toward new tasks is impaired (Nolen-Hoeksema, 1991; Levens et al., 2009). Although AMs can induce mood states such that positive AM retrieval increases post-retrieval positive mood and negative AM retrieval increases post-retrieval negative mood (Denkova et al., 2012), AMs have the additional representations of the physical, cognitive, and social environment as well as the emotional representation of the memory, and can exist independently of mood states (Tulving, 2002). Possibly related to this process, the episodic memory for such experiences is *enhanced* by the emotional salience or arousal associated with the event (Conway, 2003). Additionally, in young adults, both positively and negatively valenced memories are recalled more often (Schlagman et al., 2006) and more vividly than neutral AMs (Comblain et al., 2005).

One aim of the current study was to investigate whether the recall of emotional and neutral AMs differentially affects performance on a subsequent cognitive task. If simply recalling a memory is enough to impair performance on a subsequent cognitive task, then performance on that task should not differ following neutral and emotional autographical memory recall. If however, the emotional component is necessary for subsequent cognitive performance to be affected then performance accuracy should differ following neutral and emotionally valenced memories.

A second aim was to determine whether altering the difficulty level of a cognitive task performed immediately following AM recall would influence the memory's effect on performance of the subsequent task. The corollary to such an effect was demonstrated by Erber and Tesser (1992) in a study of the relationship between mood alteration, cognitive performance, and task difficulty, which showed that increasing the cognitive demand of a task decreased ratings of both positive and negative emotions experienced during a preceding mood induction. In this study participants underwent either negative or positive mood induction by watching a video clip and then completed either simple or difficult math problems. Solving the difficult problems—but not the simple problems—resulted in a decrease in the self-reported ratings of both positive and negative mood. These results suggest that increasing the cognitive demand of a task can reduce the intensity of an emotional experience.

We aimed to determine whether increasing the cognitive demand of an experimental task might reduce the capacity for emotional AM recall to degrade task performance, presumably by maintaining attention toward the cognitive task. By setting the task difficulty sufficiently high so that a dominant portion of an individual's attentional resources must be committed to maintain task performance, it is conceivable that an insufficient amount of attentional reserve can be allocated to support the contemplation of emotionally valenced AMs (Morrow and Nolen-Hoeskema, 1990). Therefore while we expected AM recall to degrade performance on a simple cognitive task performed in Experiment 1, we predicted that the increased difficulty of a task performed in Experiment 2 would reduce this effect. Our goal in this research was to contribute to the literature on emotion and cognition by determining whether emotionally valenced *AM recall* impairs performance on a subsequent cognitive task, and to assess whether increasing the task difficulty attenuates this effect. These results conceivably hold implications for patients with major depressive disorder who often are unable to interrupt ruminative ideation regarding past experiences to an extent that allows them to focus attention on other tasks (Levens et al., 2009).

The effects of recalling AMs on a simple continuous performance task that involved counting the number of t's in a letter string (Experiment 1), and a more difficult task that involved subtraction problems (Experiment 2) were examined. Participants were shown either a positively, negatively, or neutrally valenced cue word, and then were instructed to retrieve an AM related to the cue word and to focus on the memory. Following each memory retrieval the count-the-t's task or the subtraction task was performed. Our hypothesis was that recalling AMs would affect performance on a subsequent task and that the effect on performance could be altered or abolished by increasing the cognitive demand of the subsequent task.

## Experiment 1

### Materials and methods

#### Participants

Twenty medically and psychiatrically healthy (10 females) individuals participated in the study. Right-handed volunteers (as established using the Edinburgh Handedness Inventory; Oldfield, 1971) between 18 and 55 years of age were recruited through media advertisements in the Washington, D.C. and Tulsa, OK metropolitan areas. Half of the participants were tested at each site, and the same experimenter conducted testing at both locations. Participants underwent a screening evaluation prior to enrollment that included medical and psychiatric history. Psychiatric health was established using the Structured Clinical Interview for DSM-IV Disorders (SCID; First et al., 2002) administered by trained research nurses with at least 0.80 interrater reliability, and confirmed via unstructured interview with a psychiatrist. The Family Interview for Genetic Studies (FIGS; Maxwell, 1992) was used to assess the family history of psychiatric disorders. Participants were also administered the two-subtest version (vocabulary and matrix reasoning) of the Wechsler Abbreviated Scale of Intelligence to determine IQ (Wechsler, 1999).

Participants were excluded if they had: (a) exposure to psychotropic or other medications likely to influence cognitive function within three weeks of testing (excepting nicotine and caffeine), (b) major medical (including endocrine) or neurological disorders, (c) a history of drug or alcohol abuse within one year or a lifetime history of alcohol or drug dependence (excepting nicotine), (d) current pregnancy (as documented by urine testing), (e) a current or past history of a major psychiatric disorder, or (f) a first-degree relative with a psychiatric disorder. After receiving a complete explanation of the study procedures, participants provided written informed consent as approved by the NIH Combined Neuroscience IRB and the Western IRB. The research was conducted in accord with APA standards for ethical treatment of participants. Participants received financial compensation for their participation.

#### Material, design, and procedure

A computerized version of the AM Test (Williams and Broadbent, 1986) was employed in the current study that had been developed previously for use during functional magnetic resonance imaging (fMRI), such that participant responses were recorded via keypad entry, in order to avoid movement associated with verbal responses (Young et al., 2012). One goal of the current study was to characterize the behavioral performance on tasks that would be used to control for nonspecific cognitive processing components encountered during fMRI studies of AM recall. Participants were presented with a total of 60 words (20 positive, 20 neutral, 20 negative) selected from Bradley and Lang's (1999) “Affective norms for English words.” All words were matched for frequency of use in spoken English. Positive and negative words were matched on arousal ratings, which ranged from 2.8 to 7.6. Neutral words ranged from 2.5 to 5.24 in arousal ratings. Valence ratings of the positive and negative words were equally different from the neutral words. Negative words selected ranged in valence ranged from 1 to 2.75, while positive words ranged from 7 to 8.75, and neutral words from 4.90 to 6.10.

Table 1 presents the average ratings of the selected cue words with respect to valence and arousal, compiled from the word lists assessed and normed by Bradley and Lang (1999). A repeated measures ANOVA with the dependent variable's deviation from the neutral rating of five, arousal rating, and frequency rating for the factor experimenter-assigned valence (Positive, Negative, Neutral). There was no main effect of Frequency [*F*_(2, 55)_ = 1.39, *p* = 0.26] but there was a main effect of both arousal [*F*_(2, 55)_ = 12.4, *p* < 0.001] and deviation from neutral [*F*_(2, 55)_ = 144, *p* < 0.001]. Follow up paired *t*-tests revealed that the neutral words were less arousing than the positive or negative words [*t*_(38)_ = 5.38, *p* < 0.001], and had a smaller deviation from the mean neutral value of five than the positive or negative words [*t*s_(38)_ > 4.31, *p*s < 0.001]. Positive and negative words did not differ from each other on arousal [*t*_(38)_ = 0.47, *p* = 0.64] or in their deviation from neutral [*t*_(38)_ = 1.31, *p* = 0.20]. Stimuli were presented on a computer using e-prime (Psychology Software Tools, Inc., Sharpsburg, PA).

**Table 1:** **Characteristics of selected words**.

**Ratings according to Bradley and Lang (1999)**	**Experimenter assigned valence**		
	**Negative**	**Neutral**	**Positive**
Valence	2.25 (0.62)	5.05 (0.36)	7.73 (0.39)
Arousal	5.78 (1.22)^*^	4.10 (0.67)	5.58 (1.38)^*^
Frequency	92.3 (97.2)	102 (93.1)	92.7 (123)
Deviation from Neutral (5)	2.75 (0.62)^*^	0.05 (0.26)	2.73 (0.59)^*^

Numbers in parentheses indicates one standard deviation of the mean.

* Indicates a significant difference from the neutral cue word condition at p ≤ 0.001.

Participants were presented with a cue word and instructed to press a key on the computer once they recalled a specific memory (defined as a memory for an event that occurred during a period of no longer than one day). They had 60 s to perform this task, and if after 60 s no key had been pressed, the question, “Do you have a memory?” appeared on the computer monitor with the response options Yes/No. There was no time limit to answer this question. After participants indicated whether they had retrieved a memory, a fixation-cross appeared for 5 s during which participants were instructed to attend to the memory, focusing on the details and emotions associated with the remembered event if they had retrieved a memory, or to simply relax and clear their minds during the fixation cross presentation if no memory had been retrieved.

Following the post cue-word fixation cross a distractor task was presented. Participants were presented with a letter string and instructed to count the number of times the letter “t” appeared in the letter string. Letter strings consisted of consonants presented in all capital letters and were matched in length to the cue words. Strings were visible while participants selected their response. The number of “t”s ranged from 0 to 5 and response options to the question “How many t's are there?” were “0–1,” “2–3,” “4 or more.” Participants had unlimited time to select an answer. Following this task, a fixation-cross appeared for 8 s before the next cue word was presented. Participants were instructed to “clear their minds” during this time in preparation for the next cue word. The order of cue word presentation was pseudo-random; we placed restrictions on the order of presentation to prevent sequential presentations of a particular valence category.

Following completion of the task, participants underwent an interview with the experimenter in which they were asked a set of pre-determined questions regarding their experiences during the task. These included whether the participant was actively engaged in the task, if there were any cue words that stood out as particularly difficult to recall a memory for, how difficult they found the distractor task, and whether the distractor task was effective at distracting them from the previously recalled memory.

A subset of the participants (*n* = 10) also completed mood ratings immediately prior to and following completion of the task. This was later added after an interim analysis of the data from the first 10 participants from each experiment suggested performance differences may be influenced by the emotional valence of the cue word. These scales were added to assess whether the mood sate was altered consistently by AM recall and to evaluate potential relationships between such a mood change and behavioral performance. To rate the mood state the Profile of Mood States (POMS; McNair et al., 1971) and a 10-point Visual Analogue Scale (VAS) measuring current levels of happiness, sadness, anxiety, anger, and alertness were administered.

#### Statistical analysis

Data were analyzed using SPSS 14.0. One sample t-tests were used to determine if mood ratings changed between pre- and post- task assessments. Independent samples t-tests were used to determine if the participants in Experiments 1 and 2 differed on demographic characteristics or mood ratings. A repeated measures analysis of variance (ANOVA) was performed on the factor Valence (positive, negative, neutral) and the between subjects factor Sex (male, female) for accuracy and response times. Paired samples t-tests were conducted to determine whether there were differences in accuracy or response time following the differently valenced cue words (positive, negative, neutral). A *p*-value of ≤ 0.05, two-tailed, was selected as the statistical criterion for significance. The Bonferonni correction was applied to adjust *p*-values for the effect of multiple testing. Only trials on which a memory was retrieved were included in the analysis.

### Results

Table 2 provides the demographic characteristics and mood ratings of the participants for each study. Mood ratings on the POMS and VAS did not significantly change from pre- to post-task in Experiment 1 (one sample *t*-test comparing change scores to 0, which indicates no change; *t*s_(9)_ < 1.31 *p*s > 0.22). There was no significant differences between males and females on these measures [*t*s_(8)_ < 1.82 *p*s > 0.11].

**Table 2:** **Demographic characteristics and mood ratings for participants in each Experiment**.

	**Experiment 1**	**Experiment 2**
*n [% female]*	20 [50]	20 [50]
*Age*	32.2 (12.7)	32.2 (10.1)
*WASI*	104 (13.1)	105 (8.72)
POMS TOTAL^*^		
Pre Task	−36.6 (13.7)	−40.1 (11.9)
Post Task	−35.6 (19.4)	−37.3 (12.4)
Change	1.00 (17.1)	2.80 (4.10)
VAS – HAPPY^*^		
Pre Task	6.55 (1.78)	6.40 (1.76)
Post Task	6.45 (2.59)	6.15 (2.11)
Change	−0.10 (1.66)	−0.25 (0.54)
VAS – SAD^*^		
Pre Task	0.40 (0.70)	0.45 (1.12)
Post Task	0.30 (0.67)	0.35 (1.11)
Change	−0.10 (0.32)	−0.10 (0.32)
VAS – ANGRY^*^		
Pre Task	0.60 (0.84)	0.10 (0.21)
Post Task	0.20 (0.42)	0.05 (0.16)
Change	−0.40 (0.97)	−0.05 (0.16)
VAS – ALERT^*^		
Pre Task	7.25 (1.65)	6.85 (2.29)
Post Task	6.80 (2.30)	5.65 (2.71)
Change	−0.45 (1.61)	−1.20 (2.62)
VAS – ANXIOUS^*^		
Pre Task	0.90 (0.99)	0.60 (1.15)
Post Task	0.70 (1.49)	0.39 (1.17)
Change	−0.20 (1.14)	−0.25 (0.54)

Number in parentheses indicate one standard deviation of the mean.

* Only one half of the sample contributed to this data (5 Males and 5 Females per experiment). POMS = Profile of Mood States; VAS = Visual Analogue Scale; WASI = Wechsler Abbreviated Scale of Intelligence.

Participants recalled a memory for an average of 91.5% (55 out of 60) of the cues presented. This number didn't differ across the differently valenced cues (91% for positive, 93% for negative, 90% for neutral words). Participants selected the correct response during the count the t's distractor task 75.2 ± 12.2% of the time, and took an average of 2.38 ± 0.85 s to respond. Table 3 shows the task performance following each type of memory cue. We first examined accuracy during the count the t's task. There was no main effect of, or interaction with, sex [*Fs*_(1, 18)_ < 0.228, *ps* > 0.64] The main effect of Valence was significant [*F*_(2, 38)_ = 417, *p* < 0.001]. Follow up paired *t*-tests revealed participants were more accurate following exposure to neutral cue words than to positive cue words [*t*_(19)_ = 34.6, *p*_corrected_ < 0.001]. Participants demonstrated the worst performance following exposure to negative cue words; performance was significantly lower than that following exposure to neutral [*t*_(19)_ = 22.0, *p*_corrected_ < 0.001] and positive [*t*_(19)_ = 4.79, *p*_corrected_ < 0.001] cue words.

**Table 3:** **Mean accuracy and response times for the Count the t's task in Experiment 1**.

	**Accuracy (%)**	**RTs in seconds**
Neutral	91.3 (3.93)#	2.27 (0.84)
Positive	69.8 (3.80)^*^#	2.39 (0.77)
Negative	64.5 (2.76)^*^	2.47 (0.96)

Numbers in parentheses indicate one standard deviation of the mean.

* Indicates a significant difference from the neutral cue word condition at p ≤ 0.05.

# Indicates a significant difference from the negative cue word condition at p ≤ 0.05.

We next examined latency to select a response during the count the t's task. The ANOVA for response times was not significant [*F*_(2, 38)_ = 1, 22, *p* = 0.31]. While participants responded most rapidly following exposure to neutral cue words and most slowly following negative cue words, no difference in latency approached significance (negative versus neutral cue words: *t*_(19)_ = 1.36, *p*_corrected_ = 0.57; negative versus positive cue words: *t*_(19)_ = 0.60, *p*_corrected_ = 0.91; positive versus neutral cue words: *t*_(19)_ = 1.28, *p*_corrected_ = 0.65). Power for the Experiment 1 ANOVA = 0.99.

### Discussion

Participants performed less accurately on a simple continuous performance task requiring them to count the number of t's in a letter string when this task followed AM retrieval in response to emotionally valenced cues versus neutral cues. Performance accuracy was lowest following attempts to recall a memory related to a negative cue word than following attempts to retrieve a memory prompted by either positive or neutral cue words. Performance accuracy also was reduced to a greater extent following AMs cued by positive words than following those cued by neutral words. This performance degradation demonstrates that both emotionally positive and negative memory recall can impair performance on a simple cognitive task. Mood ratings did not change significantly from pre- to post-task, suggesting that the performance differences identified were unlikely to have been attributed simply to a difference in mood state.

The specificity of our results for AM retrieval is informed by the contrasting results of the study of Siegle et al. (2002), which used a task that involved similar cue words but required participants only to rate the valence of the word. In this task the affective valence of the cue words did not differentially alter behavioral performance on a subsequent task. In their task, cue words, taken from the same word pool as that used herein, were presented and participants were instructed to read the word and indicate its valence; immediately following each trial the participants performed a Sternberg search task in which they indicated whether a target number appeared within a series of number strings. There was no difference in performance on this latter task based on the affective valence of the cue word presented. The researchers suggested that the lack of behavioral effects might have been due to the simple nature of the number detection task. However, the emotionally-valenced cognitive task we used herein significantly influenced performance on a subsequent simple task. The crucial difference between our task series and that of Siegle et al. (2002) may have been that they required participants only to identify the valence of the words, so that mnemonic processing and emotional state were not explicitly influenced, whereas participants in the present study were instructed to retrieve memories related to the cue word. This indicates that simply reading emotionally valenced words does not induce emotional arousal to an extent that is sufficient to impair performance on a subsequent task, and that generating a memory for the cue words may be a crucial factor for the observed impairments.

Another potential difference between studies may have been that the AM task we used was more likely to alter mood than the affective evaluation task employed by Siegle et al. (2002). This explanation is inconsistent with our results in the subset of participants that completed the mood ratings, suggesting mood did not change over the course of the study. However, we cannot completely rule out an effect of mood alterations as it is possible that transient emotional changes were induced in our participants that only lasted while they were contemplating their AMs and did not persist long enough to affect mood ratings collected post-task.

Furthermore, other evidence exists which suggests that alterations in the mood state alone may not account for the impairment we found on the Count the t's task. The results from Erber and Tesser (1992), who found that solving difficult but not simple math problems resulted in a decrease in self-report ratings of both positive and negative mood (in response to a preceding mood induction) suggests that a more difficult cognitive task can either terminate or distract from an experimentally induced mood state so that the task performance is not degraded. In their study the number of simple or difficult math problems solved did not differ significantly between the positive and negative induction groups. In contrast, we observed that generating AMs to emotional cues impaired performance on a relatively simple attention task, and that this effect was greatest for memories elicited using negatively-valenced cue words. This apparent difference in the results across studies suggests that with respect to performance of a simple cognitive task, the impairment induced by the recall of an emotionally evocative event may exceed that of a mood change induced by other means (as in Erber and Tesser, 1992). Nevertheless, other design differences existed across studies that also may have influenced the results [e.g., the absence of a neutral comparator condition and the time limit imposed on response in the task used by Erber and Tesser (1992)].

The relationship between subjective emotion and task difficulty identified by Erber and Tesser (1992) raises the question of whether the impact of positive and/or negative AM recall on cognitive performance may be reduced by tasks involving a relatively higher level of difficulty. To explore this hypothesis, the distractor task used in Experiment 1 was replaced by a more difficult task involving math problems in the design of Experiment 2.

## Experiment 2

### Methods

Twenty participants (10 females) who did not participate in Experiment 1, but met the same eligibility requirements were recruited for Experiment 2. Participant characteristics can be found in Table 2. The experimental task was identical to that in Experiment 1 with the following exception: instead of the Count the t's distractor task, participants were presented with subtraction problems. In all cases a two-digit number was subtracted from a three-digit number and participants were instructed to select the correct answer from three response options. Subtraction problems were of moderate difficulty, and response options were designed so that the correct answer was not readily identifiable. Participants had unlimited time to select their response to the subtraction problem. Similar to Experiment 1, 10 of the participants completed the POMS and VAS immediately prior to and after the task.

### Results

The participants in Experiment 2 did not differ significantly from those in Experiment 1 with respect to age, IQ [*t*s_(38)_ < 0.24, *p*s > 0.81], or any mood rating (either pre- or post-task or change *t*s_(18)_ < 1.02, *p*s > 0.32]. We again did not find significant changes in mood ratings on the POMS or the VAS from pre- to post-task (Table 2; *t*s_(9)_ < 1.46, *p*s > 0.18], and there was no significant difference between males and females on these measures [*t*s_(8)_ < 1.28, *p*s > 0.24]. An additional analysis of the combined participants groups from Experiments 1 and 2 also did not identify a significant change in mood between the pre- and post-task conditions [*t*s_(19)_ < 1.72, *p*s > 0.11].

Participants were able to recall a memory for an average of 87.3% (53 out of 60) of the cue words presented. This number did not differ significantly across the distinctly valenced cue words (88% for positive, 89% for negative, and 87% for neutral words). Table 4 shows the results of Experiment 2. Overall, participants selected the correct answer to the subtraction problem 90.4 ± 7.72% of the time and took an average of 9.03 ± 3.48 s to respond. As in Experiment 1, there was no main effect of, or interaction with, sex [*F*s_(1, 18)_ < 1.06, *p*s > 0.32]. The main effect of Valence approached significance for the accuracy ratings [*F*_(2, 38)_ = 2, 76, *p* = 0.076]. In contrast to Experiment 1, participants nominally were *most* accurate following exposure to negative cue words. However, the difference in accuracy between the trials that followed a negative cue and those that followed a neutral cue only reached a non-significant trend [*t*_(19)_ = 2.08, *p*_corrected_ = 0.08]. No significant difference was found for accuracy following the negative versus positive cues [*t*_(19)_ = 1.82, *p*_corrected_ = 0.25], or following positive versus neutral cues [*t*_(19)_ = 1.06, *p*_corrected_ = 0.91]. There was no difference in the response time to select an answer to the subtraction problem across distinct cue valences (ANOVA for response times [*F*_(2, 38)_ = 1.87, *p* = 0.17]; *t*s_(9)_ < 1.84, *p*s_corrected_ > 0.25). Power for the ANOVA in Experiment 2 = 0.77.

**Table 4:** **Mean accuracy and response times for the subtraction problems in Experiment 2**.

	**Accuracy (%)**	**RTs in seconds**
Neutral	88.0 (5.48)	9.98 (4.04)
Positive	90.0 (9.46)	8.89 (2.95)
Negative	93.3 (7.12)	8.22 (3.31)

Numbers in parentheses indicate one standard deviation of the mean.

Finally, we compared performance accuracy between Experiments 1 and 2. While accuracy differed between experiments following positive and negative AM cue words [*t*s_(38)_ > 9.31, *p*s < 0.001], the performance accuracy following neutral cue words did not differ significantly between the continuous performance and subtraction tasks [*t*_(38)_ = 1.26, *p* = 0.31].

### Discussion

These results indicate that a subtraction task, which differs from the continuous performance task used in Experiment 1 with respect to being more difficult and attentionally demanding, is effective at reducing or terminating impaired cognitive performance following AM recall. In contrast to the results of Experiment 1, the highest accuracy on the subtraction task was seen after participants recalled an AM to a negative cue word, although this difference only trended toward significance. Taken together these results suggest that the more difficult task reversed the detrimental effects on performance following memory retrieval for these cue words that had been observed during performance of the simpler continuous performance task. We also did not find any significant change in mood ratings on the POMS or VAS pre- to post-task.

## General discussion

The results of Experiment 2 support the hypothesis that more difficult tasks reduce the performance deficit induced by AM recall, as demonstrated by no difference in performance following AM recall in response to the differently valenced cue words on a moderately difficult subtraction task. These results contrast with those obtained in Experiment 1 where performance on a very simple task was impaired following AM retrieval cued by the same emotionally valenced versus neutral cue words.

These observations are consistent with evidence that as the attentional demand required to perform a cognitive task increases, neurophysiological activity in the limbic and medial prefrontal cortical regions that support emotional processing and AM is suppressed (Drevets and Raichle, 1998; Simpson et al., 2000; Svoboda et al., 2006). This neural mechanism of suppressing task-irrelevant background processes that compete for the attentional resources needed to optimally perform a new, more attentionally demanding task may facilitate the disengagement from AM processing (Posner, 1995). The trend toward performance accuracy being higher following negative AM cue words in Experiment 2 suggests the hypothesis that the greater the attentional demand posed by the subtraction distractor task enables healthy individuals to attenuate the negative emotions they experienced as a consequence of recalling negative AMs.

Our results conceivably may reflect an effect of rumination, a cognitive response, often following an emotionally evocative experience, where an individual repeatedly thinks about a past experience, focusing on the feelings, meanings, and consequences of that experience (Nolen-Hoeksema, 1991). Rumination has been assessed using the Ruminative Responses Scale (Nolen-Hoeksema and Morrow, 1991), which asks participants to indicate how often they engage in ruminative thoughts or behaviors when they feel sad. As the standard assessment of rumination is a self-report scale that focuses on negative moods, previous research has emphasized the effects of negatively valenced thoughts or memories without investigating the potential effects of positive ruminations on cognitive tasks. Our results suggest the hypothesis that both negative and positive rumination can affect performance in simple cognitive tasks, but that presenting a more difficult task can attenuate the competitive effects of both types of rumination (Morrow and Nolen-Hoeskema, 1990). Our experimental design did not afford a test of these hypotheses, however, as no state measure of both positive and negative rumination exists which would have allowed us to more definitively characterize this process. Instead we inferred the presence of rumination based on the self-reported statements of participants during debriefing interviews. All but three participants reported that they often continued to think about their memories during the “Count the t's” distractor task, supporting the conclusion that rumination on the previous memory occurred and that disengaging from the memory in order to complete the Count the t's task was sometimes difficult. In contrast when participants in Experiment 2 were asked whether they were able to stop thinking about the previously recalled memory in order to complete the subtraction problems, all but one participant indicated that they were able to do so. We did not, however, inquire about how or if rumination differed between AM recall following neutral versus emotional cue words. Understanding how these ruminations are similar or different could further help to elucidate how ruminative processes are able to disrupt cognitive performance.

Although the mood ratings did not change significantly during task performance, we cannot refute the possibility that performance of the AM task induced a transient emotional state that contributed to the observed results. It is inevitable that recalling emotional memories elicits the originally experienced emotions to some degree (Talarico et al., 2004), and this can, in turn, interfere with subsequent task performance.

Another potential explanation for impaired performance on simple but not difficult tasks following emotional AM recall is that after emotional memory recall participants engage in regulatory strategies to modulate the change in emotional experience elicited by the memories, and that this engagement requires the allocation of substantial cognitive resources. In support of this hypothesis, previous studies have shown that as participants more effectively regulate their emotions, working memory performance decreases (Scheibe and Blanchard-Fields, 2009), and that as participants suppress “forbidden thoughts” they are quicker to give up solving anagrams (Muraven et al., 1998). This explanation of increased emotional regulation is potentially compatible with our hypothesized role of rumination, as increased rumination on emotional memories would likely activate emotion regulation strategies in healthy participants.

Our findings have implications for understanding the nature of the altered cognitive processing associated with MDD, as the tendency toward rumination on negative thoughts and feelings has been found to predict both the onset of MDD and the development of more severe and sustained depressive symptoms (Nolen-Hoeksema, 1991; Just and Alloy, 1997). Future studies should examine whether engaging these patients in challenging cognitive tasks can provide relief from negative ruminations. Siegle et al. (2007) proposed such an approach via Cognitive Control Training (CCT), and found that compared to standard treatment with medication and psychotherapy, six 35-min sessions performing selective and serial attentional tasks over the course of two weeks significantly improved depressive symptoms and reduced self-reported ruminations (Siegle et al., 2007).

One limitation of the current study was that we did not have a baseline measure of performance on the count the t's or subtraction task to give us an absolute effect; our results were obtained by comparing performance following presentation of emotional AM cue words compared to performance obtained following neutral cues. Additionally, although our cue words were matched in terms of frequency, other potentially relevant characteristics such as imageability (the ease with which a word arouses a mental image), were not controlled for, and could have potentially influenced the results. The phenomenological properties of the retrieved memories (arousal, valence, etc.) also were not examined and therefore whether the properties of the recalled memories (beyond cue valence) affected performance could not be determined. Because these properties were not measured we cannot refute the possibility that the subtraction task used in Experiment 2 itself impaired AM recall. Because AM retrieval depends on executive resources, and insufficient cognitive resources may lead to overgeneral AM retrieval (Dalgleish et al., 2007) the use of subtraction problems may have led to less emotional personal recollections in which the participants were not engaged enough in their memory recall enough to disrupt the performance on the subtraction task.

Furthermore, although our claims that the two tasks differed in perceived difficulty and cognitive demand have face validity and were supported by debriefing interviews in which nearly all participants perceived the subtraction task difficult and the Count the t's task as easy to perform, we did not systematically measure participants' perceived difficulty of the task. The two tasks clearly differed in other domains as well (most noticeably linguistic versus mathematical). Future studies should match cue words and subsequent tasks on as many dimensions as possible in order to minimize differences in task sets. Finally, although a sample size of 20 per experiment is lower than that typically involved in behavioral studies, the power for the ANOVAs was 0.99 and 0.77, for Experiments 1 and 2 respectively. Our observed power either exceeds or approaches the 0.80 power level that conventionally has been considered by statistical experts to provide sufficient power during experimental design. Therefore, it is unlikely that adding participants would further influence the direction or significance of results. Our relatively low sample size did, however, preclude us from investigating whether different participant characteristics might have mediated some of the observed results. Previous studies have found extroversion and neuroticism to mediate the valence of AMs recalled and subsequent effects on mood (Denkova et al., 2012), and emotional reactivity and family history of depression to mediate cognitive biases in recall of emotionally valenced items (Flynn and Rudolph, 2012). Future investigation into the interaction between personality factors and the effect of emotional AM recall on cognition may help to further elucidate the results found in the current study.

In summary, our results suggest that both positive and negative AM recall can affect performance on simple tasks (potentially due to rumination or employment of emotion regulation strategies on these memories), but that as the task difficulty increases, healthy humans can interrupt these processes and preserve performance accuracy. Our results may hold implications for cognitive behavioral therapeutic strategies involving patients with MDD, as increasing task difficulty may allow them to attenuate the potentially impairing effects of negative rumination on neuropsychological performance of tasks within a variety of cognitive domains.

### Conflict of interest statement

The authors declare that the research was conducted in the absence of any commercial or financial relationships that could be construed as a potential conflict of interest.
